# Needs assessment survey for enhancing United States child agricultural injury prevention capacity: Brief report

**DOI:** 10.3389/fpubh.2023.1059024

**Published:** 2023-03-27

**Authors:** Marsha Salzwedel, Bryan P. Weichelt, Rick Burke, Barbara C. Lee

**Affiliations:** National Children's Center for Rural and Agricultural Health and Safety, National Farm Medicine Center, Marshfield Clinic Research Institute, Marshfield, WI, United States

**Keywords:** child, youth, injury, prevention, farm, agricultural safety, needs assessment

## Abstract

The dissemination of childhood agricultural safety and health information and resources through organizations that farmers trust enhances implementation and the Socio-Ecological Model can help identify these organizations. However, to become effective partners in improving agricultural health and safety, organizations need to build capacity in child agricultural safety and health, thus, more information is needed about these organizations’ current practices, needs, and capacity for leadership, policy makers, and knowledge mobilization. An online survey was administered to organization leaders with an interest in child agricultural injury prevention, chosen through agricultural health and safety organization membership lists. Invitations to participate in the online survey were mailed to 95 organization leaders with three weekly reminders, resulting in participation from 50 organization leaders (53% response rate). Respondents indicated a high level of awareness of child agricultural injuries, yet few were actively engaged in injury prevention. When asked about “needs” for building capacity in injury prevention, over half (56%) identified a need for more promotion and dissemination of safety resources and strategies, including ATV safety, no extra riders on equipment, and keeping young children out of the worksite. The only topic that more than half of the organizations (54%) identified as “needing more information” was childhood agricultural injury surveillance. This assessment yielded valuable details for identifying opportunities, priorities, and topics for future collaborations and capacity building. Findings help inform national and international planning committees’ work, such as the next iteration of a US National Action Plan for Childhood Agricultural Injury Prevention, scheduled for release in 2024.

## 1. Introduction

Farms and ranches across the United States often serve as family homes, as well as dangerous occupational worksites. The presence of young children in these worksites is an ongoing public health concern, one more prevalent in agriculture than other industries, and one that continues to fuel researchers and injury preventionists across the field. There are far too many traumatic injury reports involving young children, some of whom were under the presumed supervision of a nearby adult (1–3). Beyond the news media reports, studies in the peer-reviewed literature continue to reaffirm the presence of young children in these dangerous worksites, often not working at the time of injury (4–8). While there is no central repository of child agricultural injury data in the United States, current data indicates that every day about 33 children are seriously injured and about every 3 days a child dies in an agricultural related incident (9).

For many years, the primary responsibility for ensuring children hired to work in agriculture were protected from agricultural hazards rested within the regulatory system, holding farm owners accountable to abide by child labor in agriculture regulations (10). However, children working on family farms are exempt from these regulations, and the regulations cover non-working children, leaving the protection of these children to the parents’ discretion. In addition, the age minimum for hazardous work in agriculture is age 16 versus 18 in non-agricultural industries (10). Consequently, since 2009, more youth have died working in agriculture, than in all other industries combine (9). The U. S. Department of Labor, with input from NIOSH and safety advocates, proposed long overdue updates to the regulations in 2011 and for the following 7 months heard an uproar from members of the farming community denouncing the role of government with respect to young workers in agriculture. Per a directive from President Obama, the DOL withdrew the proposed rules for children working in agricultural vocations (11). The government’s response was for United States Department of Agriculture (USDA) to promote safety education. In 2013 the USDA awarded Penn State funds to develop a clearinghouse of youth agricultural safety Curricula (12).

The brouhaha over proposed, then withdrawn, child labor in agriculture regulations updates sent a loud message that the farming community wants to manage the role of children with minimal government intrusion, with comments such as “It is really sad that government is getting involved in how we teach our kids to grow up on farms” (13). So, who and what influences farm parents’ safety practices? The economics of agriculture is a primary driver of decisions and many organizations and corporations influence those economics (14, 15). While the relative strength of influence is not known, it is believed that production systems, insurers and bankers are influential (16). Additionally, institution/organization-level agricultural-focused youth-serving organizations such as National FFA and major farm organizations (e.g., Farm Bureau) often guide decisions made by farm owners.

Ideally, efforts to ensure protection of children from agricultural injuries and deaths would be guided from the highest level of the Socio-Ecological Model (SEM)—public policy (17). However, despite the fact that agriculture is one of the most hazardous industries for adults and children alike, it is the least regulated with regard to child labor (10). Regulations that do exist have limitations because: (1) most farms have fewer than 11 employees and are exempt from OSHA regulations; (2) family farms have exemptions from child labor in agriculture regulations; (3) current regulations are outdated; (4) options for enforcing those regulations that exist are hampered by the sheer geographic dispersion of farming; and (5) regulations do not provide protections for non-working children (10, 18, 19).

**Figure 1:**
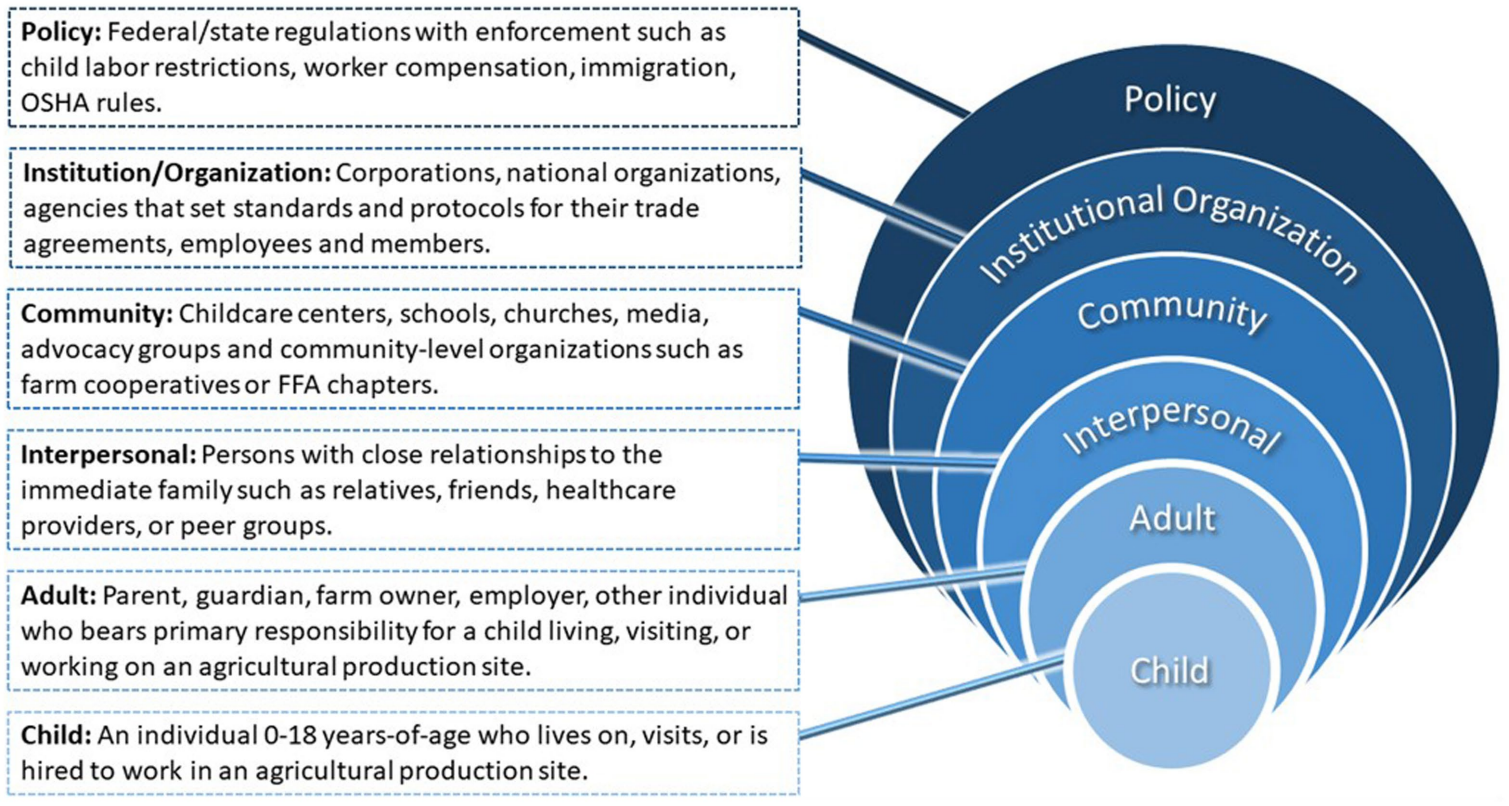
Socio-ecological model (modified for agriculture).

As regulations provide limited protections, a strategy is needed to help organizations identified in the SEM to build capacity in child agricultural safety and health. Figure 1 describes ways in which agricultural safety interventions can and should incorporate all levels of the SEM, notably the impact institution/organization and community-level interventions can have in agricultural populations (Figure 1). Increased collaboration between agricultural safety and health organizations and stakeholders, including down to the individual levels, can be a powerful tool for precipitating change.

An ideal strategy for child agricultural safety and health that engages at all levels of the SEM is described in Goal V of the 2012 Blueprint for Protecting Children in Agriculture, indicating the need to “accelerate the agricultural industry and associated organizations’ adoption of safety and health standards that protect children in agriculture” (20). As one of the leading research centers focused on United States child agricultural health and safety, the National Children’s Center for Rural and Agricultural Health and Safety (NCCRAHS) leads the efforts to assess and expand the organizational capacity of stakeholder organizations.

The purpose of this study was to assess the items (e.g., knowledge, training, resources) external agricultural safety and health stakeholder organizations need to build their capacity in child agricultural injury prevention (CAIP), and use these findings to generate recommendations for future NCCRAHS activities. The project leveraged current relationships with organization executives to reach into networks of leaders across domains of youth serving organizations, insurance companies, agricultural media, and agricultural bankers. This “reach” could also help increase the number and spectrum of groups that incorporate a focus on childhood farm safety into their ongoing systems, policies and communications with constituents and members.

## 2. Materials and methods

### 2.1. Methods

The survey instrument was developed by an interdisciplinary team of researchers and included questions related to respondent organizations’ current and planned behaviors and priorities of child agricultural injury prevention. The survey instrument was pilot tested and refined. It is available upon request. The research protocol was expedited in an ethics review by the Marshfield Clinic Research Institute Institutional Review Board, as it involved minimal risk to the participants and data was collected for research purposes using a survey. Participants confirmed their consent to participate by completing and submitting the survey. The survey sample was procured from membership lists of several key organizations, including the Childhood Agricultural Safety Network (CASN) the Centers for Agricultural Safety and Health, funded by the National Institute for Occupational Safety and Health (NIOSH), the International Society for Agricultural Safety and Health (ISASH), and the Agricultural Safety and Health Council of America (ASHCA).

These aforementioned member lists were combined with an internal NFMC contact list; duplicates were eliminated. The lists were edited to include only organizations with an interest in child agricultural injury prevention, as determined by our research team. The recipient list contained a total of 102 United States organizations. The research team reviewed the organizations and identified one individual contact from each organization to participate whom it felt could respond on behalf of the organization, typically the director or President.

The project team developed and administered an online REDCap (Research Electronic Data Capture) survey to gather information from the 102 organizations identified. Study data were collected and managed using REDCap electronic data capture tools hosted at Marshfield Clinic Research Institute (21, 22). REDCap is a secure, web-based software platform designed to support data capture for research studies, providing (1) an intuitive interface for validated data capture; (2) audit trails for tracking data manipulation and export procedures; (3) automated export procedures for seamless data downloads to common statistical packages; and (4) procedures for data integration and interoperability with external sources.

A modified Dillman approach was employed with an incentive–a $2 USD bill–with each mailed invitation (23). The hard copy invitations, which included a link to the online REDCap survey, were mailed to the identified stakeholder in each organization in December 2019. Seven of the mailings were returned unopened, with no forwarding addresses found. Three weekly reminders were emailed to all participants, as the use of a single survey link precluded identifying who had already responded. Completed responses were received from 50 of the 95 recipients, representing a 53% response rate. Our analytical sample for this paper was limited to the 50 respondents.

The survey instrument developed in REDCap used branching logic, which enabled participants to be directed to questions based on their self-identification as a CASN member and/or as a representative of a NIOSH Ag Center. If participants did not identify as either, they were directed to a set of questions for other organizations. The survey contained 20 questions, although due to branching logic, no participants were asked more than 15 questions. The survey was pilot tested with individuals from the target organizations, who were not identified as potential participants.

## 3. Results

Participants represented a good cross section of agricultural safety and health stakeholders, including academia (Researchers, Extension Specialists, and Teaching), 4-H Educator, insurance, Farm Bureau, and health and safety organizations. No responses were received from equipment manufacturers, agricultural cooperatives, bankers/lenders, and high school agricultural teachers, youth organizations or County/Regional Extension Educators/Agents. However, it is possible this is due to how the participant self-identified. For example, some organizations may be youth focused safety and health organizations, and selected as safety and health, rather than as a youth organization.

When participants were asked to select all organizations to which they were a member, ISASH was the most frequently selected (53%), followed by Other (40%), CASN (33%), NIOSH Ag Centers (31%), ASHCA (27%) and the American Society of Agricultural and Biological Engineers (24%). The “Other” category of respondent organizations included the American Association of Agricultural Education, American Psychological Association, National Safety Council, American Society of Safety Professionals, National Association of State, Public Health Vets, Occupational Safety and Health Association, National Fire Protection Association, International Rural Health Association, Farm Bureau, Rural Health Association, National Association of Community Health Centers, Grain Handling Safety Council, NCERA, and SAE (see Supplementary material for a further detailed breakdown). Most of the organizations (84%) indicated that agriculture-related injuries and fatalities to children and youth were either a major problem or somewhat of a problem. Despite the perceived seriousness of the problem, less than a third of the respondents (31%) worked on child agricultural injury prevention initiatives frequently or all the time. However, when asked to characterize their organization’s interest level in participating in CAIP activities in the next 5–6 years, 80% of participants indicated they were very interested or somewhat interested.

**Figure 2:**
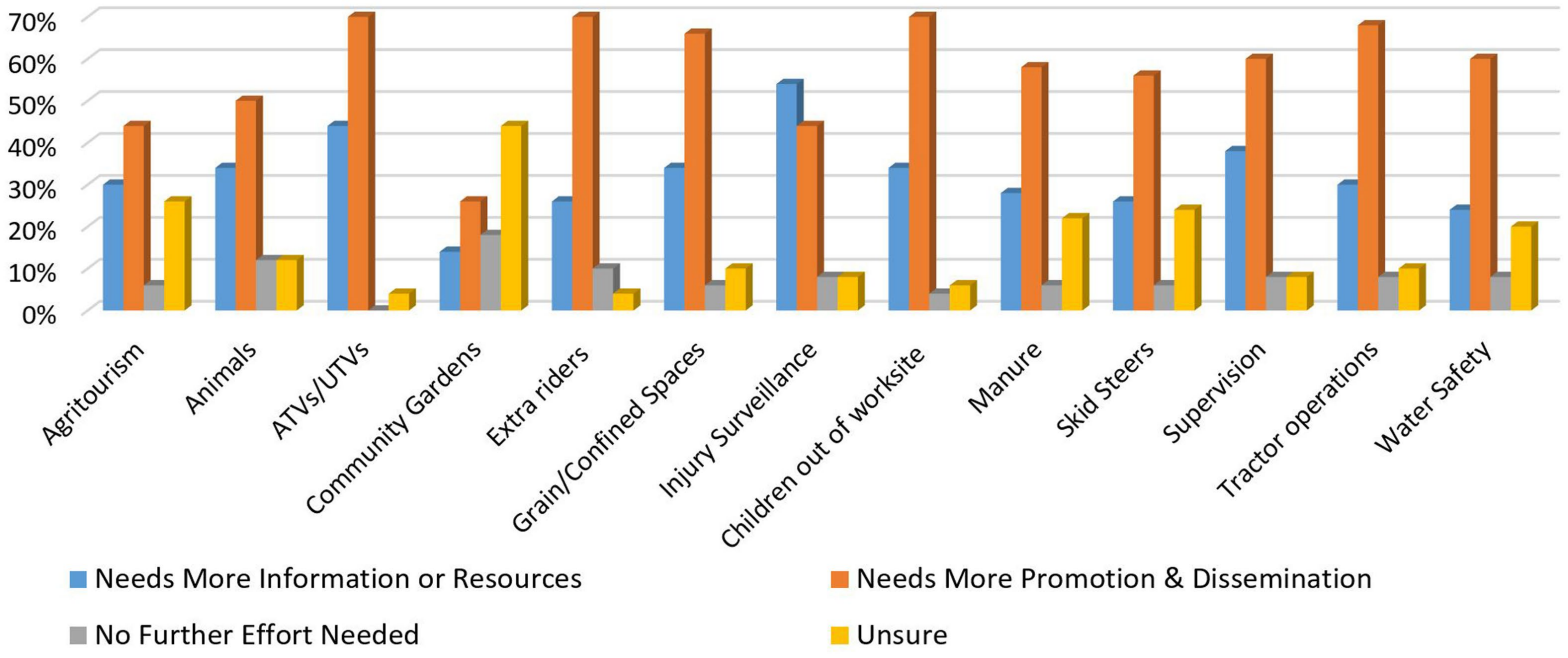
Child Ag safety and health topic needs.

Promotion and dissemination of child agricultural safety resources and tools was identified by respondents as the strongest need overall (mean = 56%), when given a list of 13 topics (Figure 2). Topics identified as needing the most promotion and dissemination were ATVs/UTVs (70%), extra riders on equipment (70%), and keeping young children out of the worksite (70%). Injury surveillance was the only topic that more than half of the respondents identified as needing more information and resources (54%).

There was also some impact of the respondents’ organization’s role in how they viewed topics of agricultural safety and health. For example, a majority of insurance respondents viewed community gardens as needing no further promotion and dissemination, while also reporting a need for greater information and dissemination for animal safety than academia or health and safety organizations. Generally, academia and health and safety organization respondents reported a greater need for more information and dissemination across all topics, while insurance and extension respondents were more conservative in several topics including youth supervision and grain/confined spaces.

Participants were also asked to list any other topics that needed to be addressed. Railroad safety, weather safety and mental health were listed by three separate participants, two participants listed hearing protection and three participants indicated a need for more Spanish resources.

**Figure 3:**
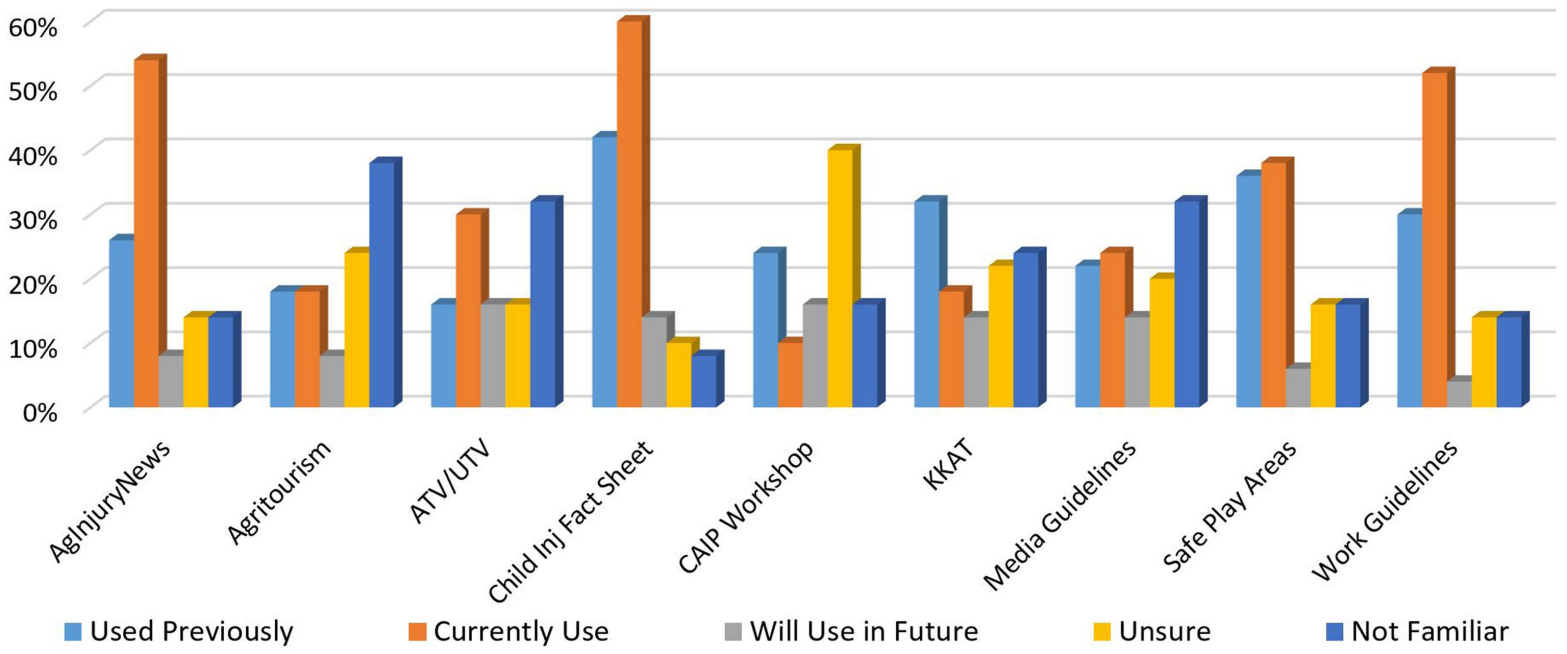
Resource usage.

The resources most identified as being in current use by survey participants are the Child Agricultural Injury Fact Sheet (60%), Ag Injury News (54%), and the Agricultural Youth Work Guidelines (52%; see Figure 3) (9, 24, 25). Participants were also invited to list any other resources they have used or will use. One participant listed the mini grant program and one participant listed Tick ID Cards. Another participant wrote: “*We have distributed nearly all of the resources available from the Children’s Center—they are a great complement to our Center’s materials and provide a conversation starter with agricultural families*” (26, 27).

**Figure 4:**
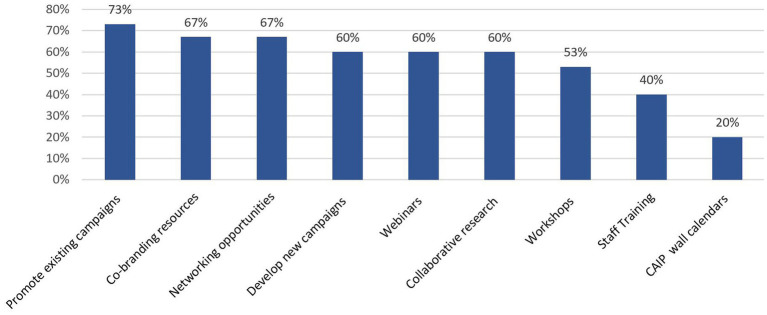
CASN member interest in CAIP activities.

Through the use of branching logic, this instrument was able to direct specific questions to CASN members to help guide their future efforts. The top three CAIP activities *CASN members* indicated an interest in were promoting existing campaigns (73%), networking opportunities (67%), and co-branding resources (67%), although several other activities were also identified by more than half of the CASN members (see Figure 4). Although asked, no participants suggested other activities. Through branching logic, the NIOSH Regional Agricultural Centers representatives’ interest in collaboration was solicited in an effort to inform future work. Results from the centers revealed interest in collaborative research projects (85%), promotional campaigns (69%) and co-branding resources (69%), while other organizations (did not self-identify as CASN or NIOSH Ag Center) were most interested in co-branding resources (60%). The” Other” activity that NIOSH Agricultural Centers indicated they were interested in was “Extension/Outreach activities.”

## 4. Discussion

This survey found a fairly high level of awareness of child agricultural injuries, yet few of respondents are actively engaged in prevention activities. While this survey did not explicitly ask participants why they were not engaged, it may be due to a lack of materials or resources, as many respondents reported promotion and dissemination as the largest need (56%). Re-surveying participants as the COVID-19 pandemic becomes less of a priority could further shed light on this discrepancy. Plans are underway to conduct a survey with CASN members in early 2023, which will assess many of these same items, allowing for comparison across timeframes and expansion to include topics such as methods of dissemination.

Another promising finding is that 60% of CASN respondents were interested in developing new public service safety campaigns. This represents ongoing interest and commitment to the field. Since these data were collected, this has sparked new program planning within CASN, including the formation of a newly appointment leadership team with members external to NCCRAHS. At the time of this writing, the leadership team is soliciting member input on topic area priorities for a new campaign.

Injury Surveillance was the only topic identified by more than 50% of all participants as needing more information or resources. Consistent with recent literature, agricultural injury surveillance lacks federal-level support, funding, and dissemination (28, 29). While some datasets and methods have surfaced and shown increased utility, much discussion is underway among stakeholders and federal agency officials regarding ideal and optimal fatal and non-fatal injury surveillance strategies for the industry (4, 28, 30–32). Meanwhile, several state-based “farm fatality reports” include child fatalities, whether the child was working at the time of the incident or not, capturing the integration of the worksite and home present on many family farms and household youth (33–36).

The findings of this study have helped better inform research strategies and dissemination efforts, including the expanded child agricultural safety and health workshop series. The research team has also refreshed the Child Agricultural Injury Fact Sheet, releasing a 2022 version (9). Respondents of this survey and anecdotal evidence from other readers have shown that this report continues to be of great interest among agricultural safety and health stakeholders and the news media. Reporters from agricultural news outlets, as well as mainstream media regularly contact NCCRAHS for injury data and information, and their articles often include quotations from staff and links to resources such as the Child Agricultural Injury Fact Sheet (37).

Still, we are fully aware of the results’ timeliness. The responses provide actionable evidence, but respondents’ interests and priorities have very likely shifted since January of 2020. Additionally, respondents appeared to report a greater need for resources and dissemination in areas more pertinent to their organizations’ goals in several topic areas such as community gardens and supervision. We recommend referencing these findings as informational and supplemental to a more current assessment of stakeholder needs. Results from the planned CASN survey will enable us to compare results with this current assessment, illuminating changes in the field over the last several years.

### 4.1. Limitations

This assessment relates largely to family farm child safety, minimally touching on hired youth or migrant, Anabaptist, Hispanic, or other underserved youth. It also revealed a lack of engagement in child agricultural injury prevention activities among survey participants, but provided limited information on the reasons for the lack of engagement. Participants were also chosen from known, existing Listservs and professional organizations that, while broad in scope and inclusion, likely did not capture all potential aspects of agricultural health and safety organizations. This survey did not include mental health as a topic area question, though one respondent did mention this in an open-ended response question; it is possible more organizations are interested or actively working in mental health but did not have the opportunity to report so. In addition, the results of this study were collected late 2019, early 2020. While these findings are useful in planning and future activities, funding allocations, and priorities, the priorities of the respondent organizations may have shifted over the past 2 years. Some of these shifts as a direct result of the global COVID-19 pandemic, others as a result of federal funding cycles. Specifically, the NIOSH Regional Agricultural Centers have undergone a competitive renewal process, submitting proposals for new projects, often in new directions from their past 5-year cycles.

### 4.2. Conclusion

This needs assessment yielded valuable information from leaders of US organizations that address safety and health in agriculture. Findings indicated that most are interested in participating in child agricultural injury prevention activities, including opportunities for future collaborations. Results also highlighted the value of several existing resources that should be maintained. In addition, the survey revealed gaps in information on certain topics and needs for further promotion and dissemination across a variety of topics. This assessment yielded critical information and is an important step in identifying opportunities and topics for future collaboration.

Agriculture remains one of the most hazardous industries in the United States, with 33 children seriously injured daily (9). The information gained in this study on the knowledge, interests and needs of external stakeholder organizations will inform NCCRAHS’ future work and collaborations, helping other organizations to build their capacity in child agricultural injury prevention.

## Data availability statement

The raw data supporting the conclusions of this article will be made available by the authors, without undue reservation.

## Ethics statement

The studies involving human participants were reviewed and approved by Marshfield Clinic Research Institute Institutional Review Board. The patients/participants provided their written informed consent to participate in this study.

## Author contributions

MS, BW, RB, and BL participated in the conception or design of the work, acquisition, analysis or interpretation of data for the work, drafting the work and revising it critically for important intellectual content, final approval of the version to be submitted and published, and all agree to be accountable for all aspects of the work in ensuring that questions related to the accuracy or integrity of any part of the work are appropriately investigated and resolved.

## Funding

Funding was provided through the National Farm Medicine Center, Marshfield Clinic Research Institute, and National Children’s Center for Rural and Agricultural Health and Safety *via* a grant from the National Institute for Occupational Safety and Health (Cooperative Agreement U54 OH009568).

## Conflict of interest

The authors declare that the research was conducted in the absence of any commercial or financial relationships that could be construed as a potential conflict of interest.

## Publisher’s note

All claims expressed in this article are solely those of the authors and do not necessarily represent those of their affiliated organizations, or those of the publisher, the editors and the reviewers. Any product that may be evaluated in this article, or claim that may be made by its manufacturer, is not guaranteed or endorsed by the publisher.
