# Supplementary material for: Needs assessment survey for enhancing United States child agricultural injury prevention capacity: Brief report
Source: Front Public Health. 2023 Mar 27;11:1059024. doi: 10.3389/fpubh.2023.1059024 (PMC10083427; doi:10.3389/fpubh.2023.1059024)
Supplement: Supplementary file 1 [file Data_Sheet_1.DOCX]

**Needs Assessment – Questions/Topics**

The purpose of this project is to gather information from key informants in order to improve the safety and health of children living on farms and working in agriculture. Your voluntary participation in this survey and responses to these questions will provide valuable insights for future planning. All responses are anonymous, and your participation is greatly appreciated.

1. How would you best describe your organization?
   - Academia (Research, Extension Specialist, Teaching)
   - County/Regional Extension Educator/Agent
   - Agricultural Employer/Farm Owner
   - Banking/Lending
   - Agricultural Cooperative
   - Equipment Manufacturer/Dealer/Sales
   - Farm Bureau
   - High School/Vocational Agricultural Educator/FFA Advisor
   - Health/Safety Organization
   - Insurance
   - Youth Organization (Other than FFA/4-H)
   - Other, please specify _________________________________
2. What is your job title/position? ____________________________________
3. Please check all the following that describe you/your organization (if any):
   - Agricultural Safety and Health Council of America Member
   - Childhood Agricultural Safety Network Member
   - International Society for Agricultural Safety and Health Member
   - NIOSH Agricultural Center Staff/Collaborator
   - American Society of Agricultural and Biological Engineers Member
   - Other national/international organization/association ___________________________________
4. How does your organization perceive agriculture-related injuries and fatalities to children and youth?

- Major problem
- Somewhat of a problem
- Minor problem
- Not a problem
- Unsure

1. How often does your organization work on Child Agricultural Injury Prevention (CAIP) initiatives/projects?

- All the time
- Frequently (at least once a month)
- Occasionally (every few months)
- Sporadically (once or twice a year)
- Rarely or never
- Unsure

1. Has your organization ever collaborated with the National Children’s Center for Rural and Agricultural Health and Safety in any child farm safety efforts?
   - Yes
   - No
   - Unsure
2. Overall, how would you characterize your organization’s interest level in participating in CAIP efforts in the next 5-6 years?
   - Very Interested
   - Somewhat Interested
   - Not Interested
   - Unsure
3. Select the answer(s) that best describe the status of these topics (in regards to children/youth):

| Topic | Needs More Information or Resources | Needs More Promotion & Dissemination | No Further Effort Needed | Unsure |
| --- | --- | --- | --- | --- |
| Keep young children out of worksite |  |  |  |  |
| Extra riders on tractors/equipment |  |  |  |  |
| Tractor operations |  |  |  |  |
| ATVs/UTVs |  |  |  |  |
| Skid Steers |  |  |  |  |
| Animals |  |  |  |  |
| Grain/Confined Spaces |  |  |  |  |
| Manure Safety |  |  |  |  |
| Water Safety |  |  |  |  |
| Agritourism |  |  |  |  |
| Community Gardens |  |  |  |  |
| Injury Surveillance |  |  |  |  |
| Supervision |  |  |  |  |

List other child farm safety or injury prevention topics and what actions you feel should be taken: __________

1. Select the answer(s) that best describe your use of the following resources:

| Resource | Used Previously | Currently Use | Will Use in Future | Unsure | Not Familiar w/ Resource |
| --- | --- | --- | --- | --- | --- |
| Youth Work Guidelines |  |  |  |  |  |
| Safe Play Areas on Farms Guidelines |  |  |  |  |  |
| Agritourism Guidelines |  |  |  |  |  |
| AgInjuryNews |  |  |  |  |  |
| Child Ag Injury Fact Sheet |  |  |  |  |  |
| Child Ag Injury News Clippings |  |  |  |  |  |
| Keep Kids Away from Tractors Posters |  |  |  |  |  |
| ATV Safety Posters/ Rack Cards |  |  |  |  |  |
| Media Guidelines |  |  |  |  |  |
| Child Ag Injury Prevention Workshop |  |  |  |  |  |

List any other resources that you have used or will use: __________________________

**CASN Members**

1. In which of the following activities would your organization be interested in participating? *Check all that apply*

- Promotion of existing campaigns (Keep Kids off Tractors, ATV, Media Guidelines)
- Developing new campaigns (e.g. Skid Steer Safety)
- Co-branding resources
- Child safety printed wall calendars - branch logic– 1b
- Staff Training
- Webinars
- Workshops
- Collaborative research
- Networking Opportunities (e.g. meetings, conferences)
- Other, please specify__________________________________

1b. Would your organization be willing to help offset the cost of child safety calendars by any of the following:

C*heck all that apply*

- Paying to host a calendar page (monthly message/theme)
- Paying for calendars
- Paying for shipping
- Other, please specify __________________________________

1. Which of the following do you see as the Child Agricultural Safety Network’s roles/responsibilities? *Check all that apply*
   - Promoting/disseminating CAIP information, including press releases and resources
   - Disseminating child injury incidents
   - Providing/facilitating partnerships and collaborations
   - Leading CAIP initiatives and campaigns (existing)
   - Leading the development of new CAIP materials and campaigns
   - Providing opportunities for collaborative outreach/education initiatives (e.g. workshops, webinars)
   - Providing opportunities for collaborative research
   - Other, please specify __________________________________
2. Do you have other ideas/thoughts on what the Childhood Agricultural Safety Network could/should be doing?

- Yes (branch logic – if yes, please explain/describe)
- No
- Unsure

**NIOSH Ag Centers**

1. Does your center have any child agricultural safety or injury prevention projects/initiatives currently?

- Yes (branch logic – if yes, please explain/describe)
- No
- Unsure

1. If funding was provided, would your center be willing to partner with NCCRAHS to carry out any of the following CAIP activities? *Check all that apply*
   - Research project (1-2 years)
   - Promotional campaign of CAIP materials
   - CAIP Workshop
   - Learning labs or other meeting adjunct activity (e.g. add to ASHCA Summit, ISASH Conference)
   - Co-branding/promotion of resources
   - Other, please specify __________________________
2. Do you currently have topics, resources or ideas for CAIP activities/projects that you would like NCCRAHS to assist with?

- Yes (branch logic – if yes, please explain/describe)
- No
- Unsure

**Other Organizations**

1. Do you have any child agricultural safety or injury prevention projects/initiatives currently? (branch logic – if yes, please explain/describe)
2. Is your organization interested in addressing child agricultural safety or injury prevention in the next few years? (branch logic: yes = a, b, no = c)
3. If funding was available, would your organization be willing to partner with NCCRAHS to carry out any of the following CAIP activities? *Check all that apply*

- Research project
- Promotional campaign of CAIP materials
- CAIP Workshop
- Learning labs or other meeting adjunct activity (e.g. add to ASHCA Summit, ISASH Conference)
- Co-branding/promotion of resources
- Other, please specify __________________________

1. Do you/your organization currently have topics, resources or ideas for CAIP activities that you would like NCCRAHS to assist with?
   - - Yes (branch logic – if yes, please explain/describe)
     - No
     - Unsure
2. If you are unable to work on CAIP initiatives, would you be able/willing to assist NCCRAHS with their work in CAIP by providing any of the following? *Check all that apply*

- Resources
- Networks (introduce and/or help recruit partners, participants, etc.)
- Other, please specify ___________________________

Please add any additional comments: _____________________________________________

Thank you for your helping us with this important project. If you have any questions, comments or feedback that you would like the National Children’s Center to respond to, please contact Marsha Salzwedel, EdD. Email: [salzwedel.marsha@marshfieldresearch.org](mailto:salzwedel.marsha@marshfieldresearch.org) or phone: 1-800-662-6900, ext. 8.
