# Supplementary material for: Needs assessment survey for enhancing United States child agricultural injury prevention capacity: Brief report
Source: Front Public Health. 2023 Mar 27;11:1059024. doi: 10.3389/fpubh.2023.1059024 (PMC10083427; doi:10.3389/fpubh.2023.1059024)
Supplement: Supplementary file 2 [file Data_Sheet_2.docx]

Supplemental Figure 1. – Organizational Memberships


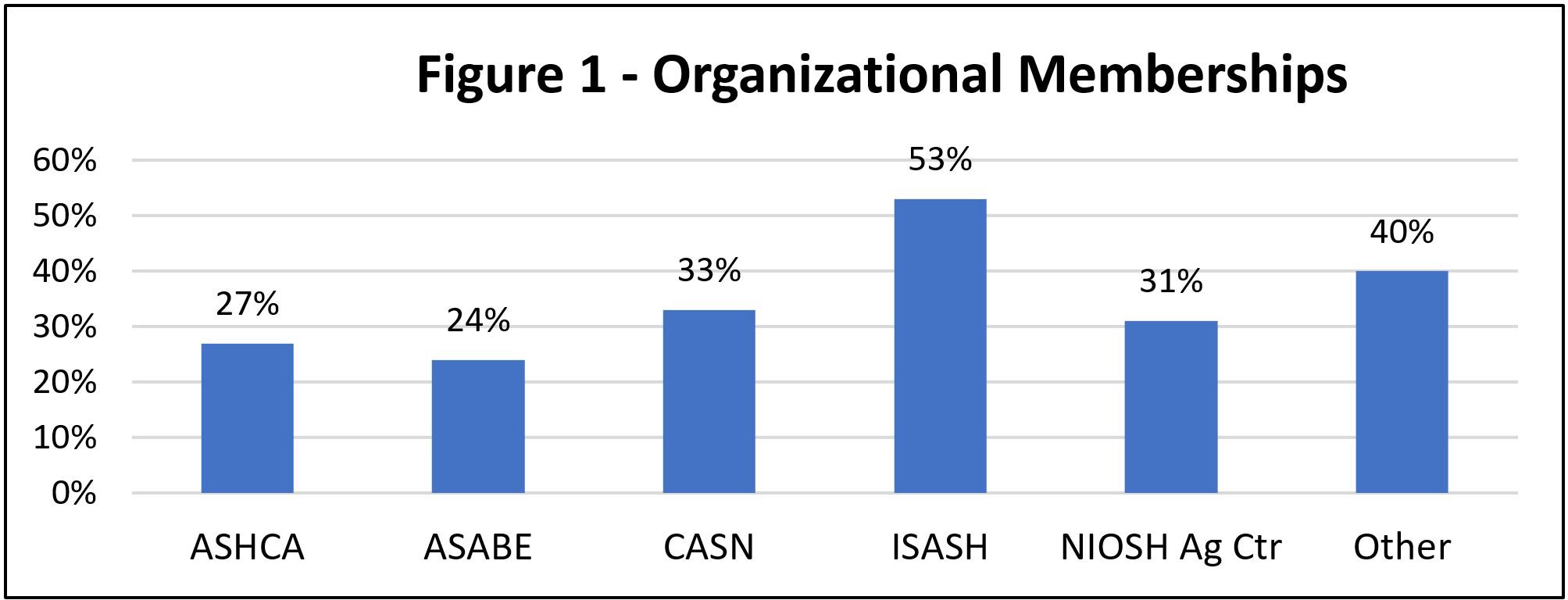


Explanation of Terms and Acronyms

ISASH – International Society for Agricultural Health and Safety <https://isash.org/>

CASN – Child Agricultural Safety Network <https://cultivatesafety.org/casn/>

NIOSH Ag Ctr – National Institute for Occupational Safety and Health Centers for Agricultural Safety and Health <https://www.cdc.gov/niosh/oep/agctrhom.html>

ASHCA – Agricultural Safety and Health Council of America <http://ashca.org/>

NCERA – North Central Extension and Research Activity <https://www.controlledenvironments.org/>

SAE – Supervised Agricultural Experience <https://thecouncil.ffa.org/sae/>
